# Subcellular Localization and Dynamics of the Bcl-2 Family of Proteins

**DOI:** 10.3389/fcell.2018.00013

**Published:** 2018-02-13

**Authors:** Nikolay Popgeorgiev, Lea Jabbour, Germain Gillet

**Affiliations:** ^1^Université de Lyon, Centre de Recherche en Cancérologie de Lyon, U1052 Institut National de la Santé et de la Recherche Médicale, UMR Centre National de la Recherche Scientifique 5286, Université Lyon I, Centre Léon Bérard, Lyon, France; ^2^Hospices Civils de Lyon, Laboratoire d'anatomie et Cytologie Pathologiques, Centre Hospitalier Lyon Sud, Pierre Bénite, France

**Keywords:** Bcl-2 family, subcellular localization, mitochondria, endoplasmic reticulum, nucleus

## Abstract

Bcl-2 family proteins are recognized as major regulators of the mitochondrial pathway of apoptosis. They control the mitochondrial outer membrane permeabilization (MOMP) by directly localizing to this organelle. Further investigations demonstrated that Bcl-2 related proteins are also found in other intracellular compartments such as the endoplasmic reticulum, the Golgi apparatus, the nucleus and the peroxisomes. At the level of these organelles, Bcl-2 family proteins not only regulate MOMP in a remote fashion but also participate in major cellular processes including calcium homeostasis, cell cycle control and cell migration. With the advances of live cell imaging techniques and the generation of fluorescent recombinant proteins, it became clear that the distribution of Bcl-2 proteins inside the cell is a dynamic process which is profoundly affected by changes in the cellular microenvironment. Here, we describe the current knowledge related to the subcellular distribution of the Bcl-2 family of proteins and further emphasize on the emerging concept that this highly dynamic process is critical for cell fate determination.

## Introduction

Intracellular compartmentalization is a fundamental feature of eukaryotic cells. It allows the physical segregation and simultaneous execution of distinct biochemical processes within the same cell. In fact, proper subcellular targeting and distribution are critical for the function of intracellular proteins. B-cell lymphoma 2 (Bcl-2) family of proteins are acknowledged as major regulators of the intrinsic pathway of the programmed cell death type 1 commonly known as apoptosis. Bcl-2, the founding member of the family, was discovered more than 30 years ago in the course of a study on the t(14;18) genomic translocation. Translocations between chromosome 18 (q21) and chromosome 14 (q32) are frequently observed in human B-cell follicular lymphomas and can result in the relocation of the *bcl2* open reading frame downstream of the enhancer promoter region of the *igh* heavy chain immunoglobulin gene (Tsujimoto et al., 1984, 1985). This translocation results in upregulation of *bcl2* gene expression (Cleary et al., 1986). Previous observations on other B-cell lymphomas such as Burkitt's lymphoma or mantle cell lymphoma, in which similar translocations led to the overexpression of oncogenes such as *c-myc* or *cyclin d1*, suggested that *bcl2* was another oncogene inducing uncontrolled proliferation. However, soon after this discovery, Vaux and colleagues demonstrated that Bcl-2 could sustain cell survival of lymphoid cells in absence of Interleukin-3, establishing Bcl-2 as the founding member of a new class of oncoproteins that inhibited cell death instead of promoting cell proliferation (Vaux et al., 1988). Since the discovery of Bcl-2, extensive work in mammalian cells as well as in other animal models, such as nematode and drosophila, uncovered a family of structurally related proteins involved in the control of apoptosis, reviewed in Delbridge et al. (2016). Indeed, Bcl-2 family members are globular proteins mainly composed of α-helices and characterized by conserved Bcl-2 homology (BH1-4) domains. On this basis, three Bcl-2 family subgroups have been identified: (1) the anti-apoptotic multidomain members (e.g., Bcl-2, Bcl-xL, and Mcl-1) containing all four BH domains, (2) the pro-apoptotic multidomain members (e.g., Bax, Bak, and Bok) containing three BH domain (BH1-3) and (3) the pro-apoptotic BH3-only members containing the sole BH3 domain (e.g., Bad, Bid, and Bik). In addition, numerous Bcl-2 proteins contain a hydrophobic transmembrane anchoring (TM) domain at the C-terminus end allowing them to localize to intracellular membranes. At the level of the mitochondria, proapoptotic Bax and Bak can form oligomers and induce the mitochondrial outer membrane permeabilization (MOMP), which is considered as a point of non-return in the execution of apoptosis. By direct binding to Bax and Bak, anti-apoptotic Bcl-2 proteins inhibit the MOMP. In addition, the BH3-only proteins control this process by direct activation of Bax and Bak or by repressing the anti-apoptotic Bcl-2 family members (Youle and Strasser, 2008). In fact, it rapidly became clear that Bcl-2 proteins can also localize to other organelles including the endoplasmic reticulum (ER), the Golgi apparatus, the nuclear outer membrane (NOM) or the nucleus itself. At the level of these different intracellular membranes, Bcl-2 proteins did not only control the MOMP from a distance, but also participated in a number of non-apoptotic processes. However, the implications of these “moonlighting” functions in physiological or pathological situations have been enlightened only recently.

Here, we discuss the current knowledge about the mechanisms addressing Bcl-2 family proteins to intracellular membranes and emphasize the emerging concept that this highly dynamic process is critical for cell fate determination.

## Subcellular action range of the Bcl-2 family of proteins

Bcl-2 family members are mainly known as regulators of the mitochondrial outer membrane integrity. However, the exact distribution of Bcl-2 itself was already a matter of debate in the early 90's. Indeed, initial studies reported that Bcl-2 could be found at the level of the cytosolic leaflet of intracellular membranes. However, experiments conducted by Korsmeyer lab suggested that Bcl-2 is in fact located in the mitochondrial inner membrane (MIM) (Hockenbery et al., 1990). These observations were somehow overlooked since it was shown later on that Bcl-2 possesses a C-terminus transmembrane (TM) domain (as discussed below), addressing this protein to the MOM. However, subsequent work showed that Bcl-2 could also control the activity of MIM proteins such as Cytochrome c oxidase Va and Cyclophilin D through direct interactions. This apparent contradiction might be explained by the actual localization of Bcl-2 at the contact points between the internal and the external mitochondrial membranes (Nakai et al., 1993; Nguyen et al., 1993). Interestingly, Bcl-2 was not the only protein detected at the MIM. Indeed, seminal investigations conducted by Jonas and colleagues revealed that Bcl-xL protein localizes at the MIM in hippocampal neurons. In this compartment, Bcl-xL directly binds to the β-subunit of the F_1_F_O_ ATP synthase, increasing its activity and allowing optimal synaptic transmission. Mcl-1 is another Bcl-2 homolog found to operate at the level of different mitochondrial compartments. At the level of the MOM, Mcl-1 inhibits apoptosis, whereas at the level of the mitochondrial matrix, it fosters mitochondrial bioenergetics. Of note, Mcl-1 matrix localization requires proteolytic cleavage of its N-terminal mitochondrial targeting sequence (Perciavalle et al., 2012).

**Figure 1:**
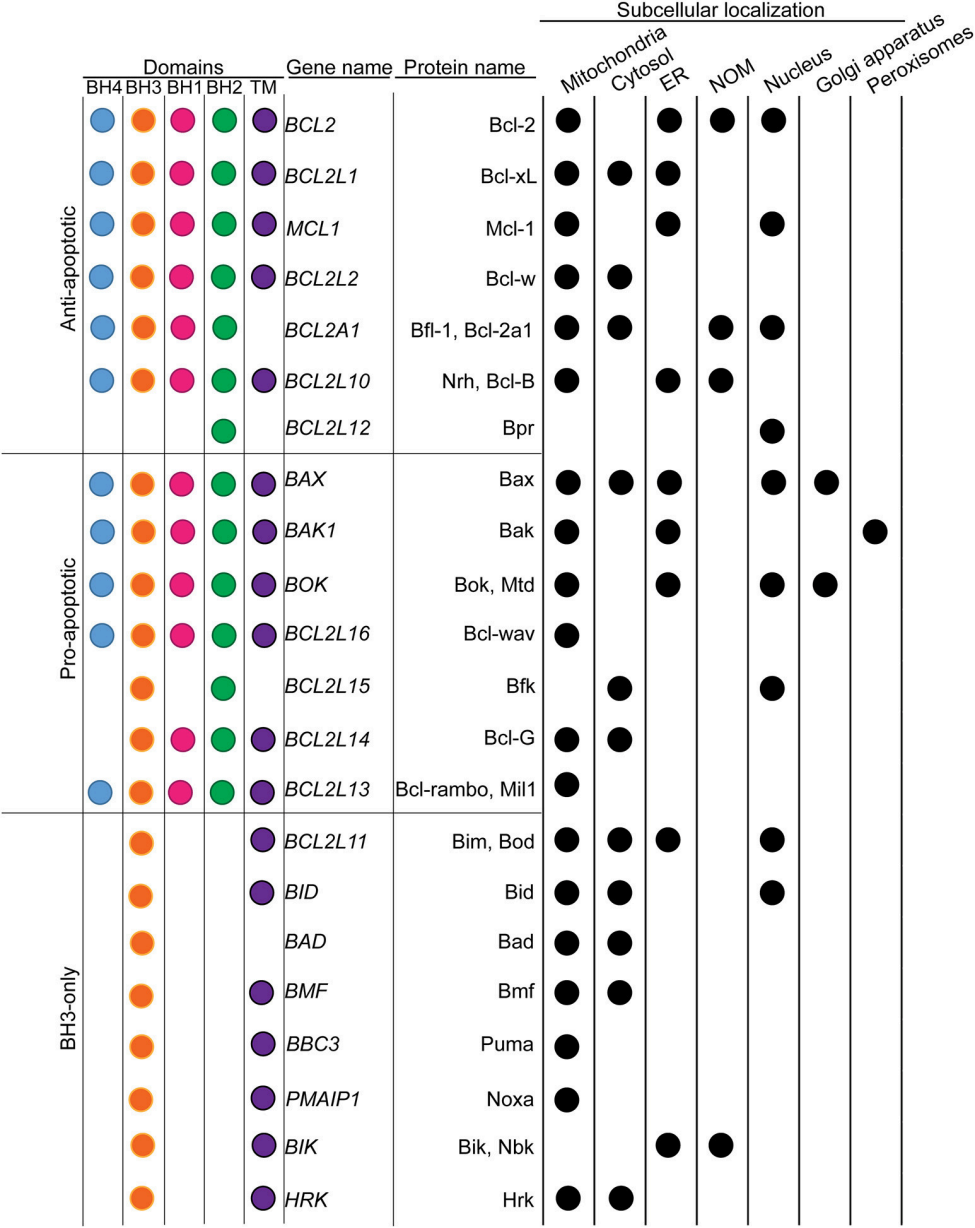
Classification of Bcl-2 family of proteins in respect to their subcellular localizations. Bcl-2 family members were classified based on their implication in the MOMP. The presence of conserved Bcl-2 homology (BH) and transmembrane (TM) domains were indicated with color dots. The main subcellular localizations were presented in the table on the right. Black dots indicate the presence of the protein to the corresponding subcellular localization.

During the course of these studies, it also appeared that several Bcl-2 homologs were in fact ER-residents. This includes both multidomain anti-apoptotic (Bcl-2, Bcl-xL, Mcl-1, and Bcl-2l10) (Krajewski et al., 1993; Yang et al., 1995; Aouacheria et al., 2001; White et al., 2005) and pro-apoptotic proteins (Bax, Bak and Bok) (Zong et al., 2003; Schulman et al., 2013) as well as BH3 only proteins (Bim, Bik) (Germain et al., 2002; Morishima et al., 2004; Figure 1). Thus, at the level of the ER, Bcl-2 proteins not only control MOMP in a remote fashion, but also orchestrate additional non-apoptotic pathways through direct binding with Ca^2+^ transporters and mediators of the Unfolded Protein Response (UPR).

Interestingly, Bcl-2 proteins were found to translocate inside the nucleus as well. In the case of Bcl-2 itself, such localization depends on the phosphorylation status of Thr56. Nuclear Bcl-2 appears to take part in a multiprotein complex comprising CDK1, PP1 and Nucleolin (Barboule et al., 2005, 2009). However, the mechanisms governing Bcl-2 nuclear translocation as well as their actual biological relevance remain poorly understood.

Finally, Bcl-2 family members may also possess some “exotic” subcellular localizations including the Golgi apparatus (Dumitru et al., 2012), the lysosomes (Guan et al., 2015) and the peroxisomes (Hosoi et al., 2017).

Their possible localization at the level of these diverse internal membranes is an emerging concept. Actually, it might illustrate the dynamics of Bcl-2 family of proteins action range and their pleiotropic functions inside the cell.

## Docking of the Bcl-2 family of proteins to intracellular membranes

The first evidence of the membrane localization of Bcl-2 arose from the work of Cleary and colleagues. By performing subcellular fractionation and immunofluorescence experiments on lymphoid cell lines, they demonstrated that Bcl-2 was associated almost exclusively with cellular membranes, as no Bcl-2 was detected in the cytosolic fraction, whereas only a minor fraction was detected in the nucleus (Chen-Levy et al., 1989). Furthermore, the Bcl-2 protein was recovered almost solely in a detergent-rich phase, confirming its membrane anchoring properties. Sequence analyses identified a 23 amino acid-long hydrophobic sequence at the C-terminus end, referred to as transmembrane (TM) domain (Chen-Levy et al., 1989). With the exception of Bfl-1, in which the TM domain is less well defined, all mutidomain Bcl-2 homologs possess a similar TM domain. Of note, this domain seems to be conserved throughout evolution since it is found in numerous Bcl-2 homologs from distantly-related metazoan species (Quinn et al., 2003; Tan et al., 2007). The TM domain is organized as a hydrophobic α-helix, which was initially proposed to contribute to the docking of Bcl-2 into membranes. Indeed, when deleted from its C-terminal hydrophobic domain, Bcl-2 becomes cytosolic (Nguyen et al., 1993). Interestingly, this observation is not restricted to Bcl-2 itself but appears to be a genuine rule for almost all multidomain Bcl-2 homologs. However, the sole presence of a hydrophobic C-terminal region does not explain why closely related Bcl-2 homologs exhibit different subcellular distributions. For instance, Bcl-2, and Bcl-xL both are anti-apoptotic and possess a hydrophobic α-helix. Nonetheless, Bcl-xL is mainly found in the mitochondria -although being in part cytosolic and ER resident- whereas Bcl-2 is found at the level of the ER, the NOM, the nucleus and the mitochondrial membranes (Krajewski et al., 1993; Akao et al., 1994; Kaufmann et al., 2003). By analyzing the TM domains of these proteins, Borner and colleagues discovered that the apparent localization differences came from the residues flanking the hydrophobic α-helix. Using targeted mutagenesis, they demonstrated that, in the Bcl-xL sequence, the presence of at least two positively charged residues—located at each side of the hydrophobic α-helix—is critical for Bcl-xL insertion into the MOM (Kaufmann et al., 2003). Indeed, such residues are absent in the Bcl-2 TM domain, which presumably allows Bcl-2 to interact with other intracellular membranes. In this respect, TM swapping between Bcl-xL and Bcl-2 resulted in inversion of their subcellular localization. Finally, Borner and colleagues demonstrated that the TM domain of Bcl-xL represents a *bona fide* MOM targeting signal since it is sufficient to address recombinant GFP to the MOM, in contrast to that of Bcl-2 that addresses the GFP to both mitochondrial and ER membranes (Kaufmann et al., 2003).

It is worth to note that the TM domain of Bcl-xL also contributes to homodimerization (Jeong et al., 2004). Indeed, substantial amounts of Bcl-xL are found in the cytosol where it forms homodimers through its TM domain (Jeong et al., 2004). Moreover, the deletion of the last two C-terminal amino acids (namely R233 & K234) significantly decreased homodimer formation, whereas the deletion of the last four amino acids completely abolished this process (Jeong et al., 2004).

Bcl-xL is not the only Bcl-2 homolog residing in the cytosol. For example in healthy cells, Bax is mainly cytosolic but becomes mitochondrial upon cell death induction (Hsu et al., 1997). This is due to the fact that under non-stressful conditions, the TM domain (α9 helix) of Bax is sequestered within its hydrophobic surface groove (Wolter et al., 1997). Upon stress induction, Bax α9 helix unmasks and anchors the protein to the MOM. Interestingly, in such conditions, Bax ΔTM deletion mutant can still translocate to the mitochondria, suggesting that additional internal signals contribute to the proper subcellular localization of Bax (Er et al., 2007). It has been proposed that the N-terminus α1 helix can directly address activated Bax toward the mitochondrial surface. Indeed, Vallette and colleagues demonstrated that Bax α1 helix is capable of docking red fluorescent protein (RFP) to the mitochondria (Cartron et al., 2003). It was also shown that the α5 helix was necessary and sufficient for Bax oligomerization and MOMP induction (George et al., 2007). Additional studies further demonstrated that fusions of GFP with Bax fragments containing the α5, α6, and/or α9 helices, either alone or within the α5α6 or α5-α9 constructs, all localize to the mitochondria. Specifically, α5 and α6 helices not only bind intrinsically to the MOM, but also show high membrane destabilization properties. On this basis, mitochondria-targeted cytotoxic agents, referred to as “poropeptides,” could be developed in our lab from natural Bcl-2-like membrane-active segments such as Bax α5 and α6 helices (Valero et al., 2011).

Bcl-2 α1 helix, also referred to as Bcl-2 BH4 domain, may also participate in subcellular targeting. For instance, the human FK506-binding protein 38 (FKBP38), a mitochondrial chaperone, is able to interact with and effectively target Bcl-2 to the mitochondria (Shirane and Nakayama, 2003). Importantly, a Bcl-2 mutant lacking the BH4 domain fails to interact with FKBP38 and is translocated to the nucleus, where it promotes apoptosis (Portier and Taglialatela, 2006).

Bcl-2 BH4 domain also interacts with the inositol trisphosphate receptor (IP3R) at the level of the ER (Monaco et al., 2012a). This domain interacts with the MTDII of IP3R and regulates its activity. Interestingly, subtle differences in the amino acid composition of Bcl-2 and Bcl-xL BH4 domains explain—at least partly—these distinct localizations. Bultynck and colleagues identified Lys17 in the BH4 domain of Bcl-2 as critical for IP3R binding (Monaco et al., 2012b). Interestingly enough, the Bcl-xL BH4 domain, which lacks Lys17, binds instead to the mitochondrial voltage dependent anion channel (VDAC). In fact, Bcl-xL α1 helix is able to target GFP to the mitochondria (McNally et al., 2013). Moreover, studies by Cheng and colleagues suggested that VDAC2, a minor VDAC isoform, is critical for Bak localization to the mitochondria (Cheng et al., 2003). Indeed, VDAC2 interaction with Bak was shown to prevent Bak oligomerization, compromising its pro-apoptotic activity. This model was further developed by the team of Hajnóczky who showed that VDAC2 is critical for the exclusive recruitment into the MOM of newly synthesized Bak molecules (Roy et al., 2009).

Finally, Bcl-2 proteins are major trans-regulating elements that control Bax subcellular localization. Indeed, BH3-only proteins such as Bim, Puma, or the pro-apoptotic Bcl-2 homolog tBid, have been suggested to mediate the insertion of cytosolic Bax into the OMM by directly interacting with Bax (Desagher et al., 1999; Letai et al., 2002; Kuwana et al., 2005; Kim et al., 2009). In this respect it has recently been shown that, with the notable exception of Bad, all canonical BH3-only proteins possess a functional C-terminus TM domain (Wilfling et al., 2012; Andreu-Fernández et al., 2016).

## Mitochondria-to-cytosol bax and bak trafficking

The observations that upon apoptotic stress Bcl-xL and Bax translocate from the cytosol to the mitochondria suggested that their localization is not static but rather reflects dynamic processes depending on the cellular status and microenvironment. Indeed, Youle and colleagues (Wolter et al., 1997) while exploring the localization of Bcl-2, Bcl-xL, and Bax in live cells using GFP fusion proteins, revealed that Bax translocates from the cytosol to the mitochondria upon apoptosis induction. Confocal microscopy followed by photobleaching experiments confirmed the cytosolic localization of GFP-Bax, contrary to organelle-bound GFP-Bcl-2. Apoptosis induced by Staurosporine (STS) led to significant changes of GFP-Bax distribution, as illustrated by the switch from a diffuse to a punctate mitochondrial fluorescence pattern. Importantly, they noticed that GFP-Bax redistribution took place before apoptotic cells shrinkage. Moreover, deletion of the C-terminal hydrophobic tail both prevented GFP-Bax redistribution and suppressed its death-promoting activity, confirming the importance of the TM domain. Nevertheless, the sole targeting of Bak or Bax to the MOM, does not necessarily imply that these proteins are spontaneously active once the TM regions are membrane-integrated. Activation of Bak and Bax appears to request some additional conformational changes, such as (1) α1 helix exposure, (2) transient BH3 exposure, (3) protection of the membrane-embedded Bcl-2 core, and (4) increased proximity of embedded monomers (George et al., 2007). Overall, these data raised the following major issue: “if Bax resides on the mitochondrial membranes upon apoptosis induction, could it be that it diffuses back to the cytosol, and thus how, when the microenvironment returns to normal physiological conditions?”

A few years later, Youle and colleagues directly demonstrated that the retrotranslocation of Bax from the mitochondria to the cytosol could be induced by the anti-apoptotic protein Bcl-xL (Edlich et al., 2011). The original purpose of their work was to understand how anti-apoptotic Bcl-2 proteins controlled Bax localization without directly interacting with cytosolic Bax. To this aim, they hindered conformational changes involving the α1 and α2 helices of Bax, which encompass the BH3 domain, to check their contribution to Bax activation. Indeed, such conformational changes were supposed to be required for Bax integration into the MOM. Thus, to maintain Bax inactive, residues F30 and L63 were replaced with cysteines, leading to the formation of intramolecular disulphide bonds between α1 and α2, along with other substitutions. Such intramolecular tethers were proven to maintain the protein in an inactive conformation, similar to that of wild type (WT) Bax. Caspase 3/7 apoptosis assays showed that these tethers compromise the activation of Bax by BH3-only proteins and affect its regulation by Bcl-xL, which must be added to their capacity to interfere with detergent-induced Bcl-xL binding. Furthermore, tethered Bax appeared to locate to the mitochondria, which was rather unexpected since Bax was supposed to be cytosolic, when maintained inactive. In fact, Fluorescence Loss In Photobleaching (FLIP) experiments revealed that Bcl-xL increased mitochondrial Bax off rates, without any detectable competition between Bax and Bcl-xL for MOM binding, proving that Bax effectively retrotranslocates from the mitochondria to the cytosol, when inactivated.

**Figure 2:**
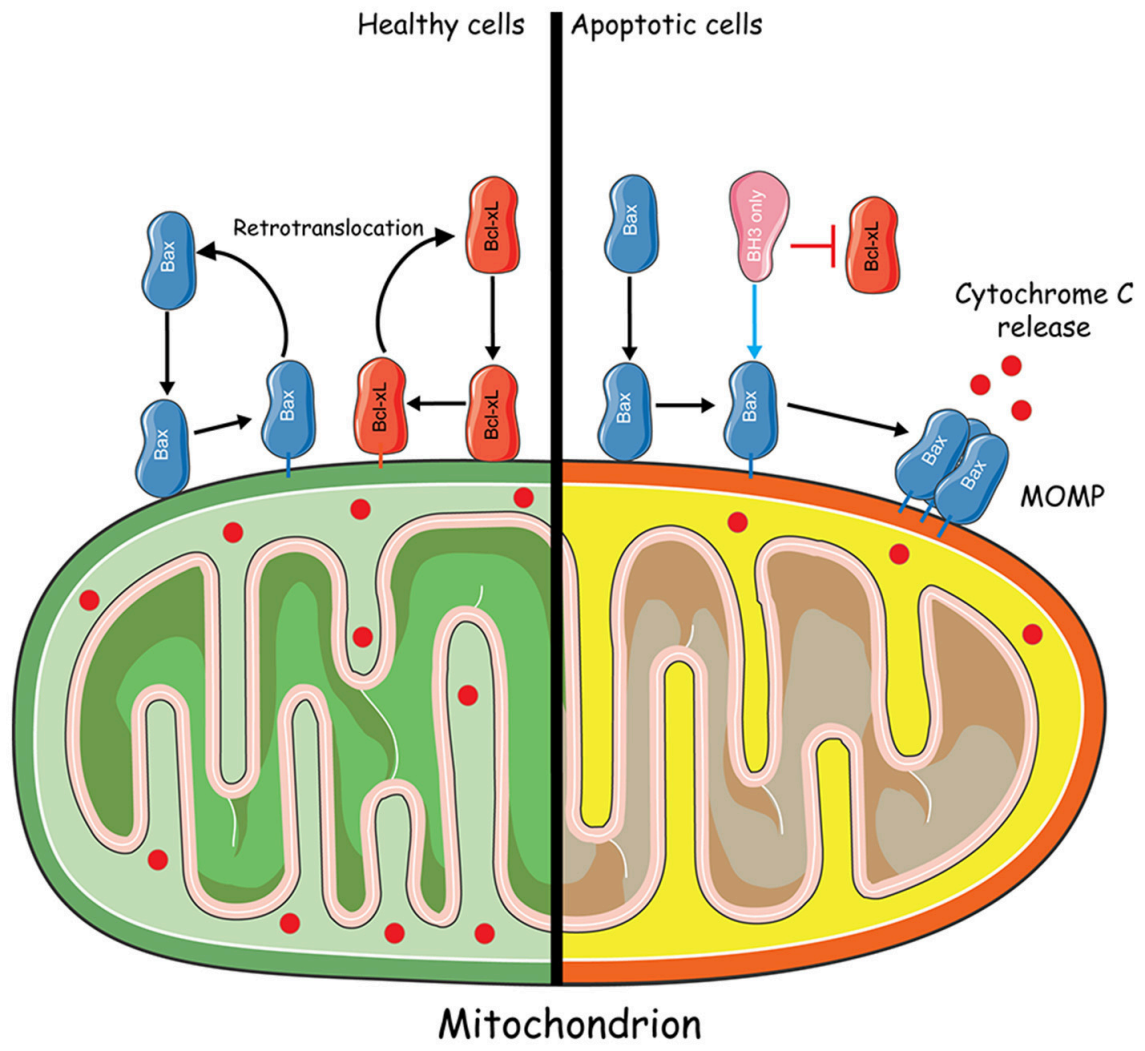
Mitochondria-to-cytosol Bax trafficking. In healthy cells, Bax constantly translocates from the cytosol to the mitochondria. Retrotranslocation of Bax from the mitochondria to the cytosol could be induced by the anti-apoptotic protein Bcl-xL through the formation of a heterocomplex. In apoptotic conditions, Bcl-xL is inhibited by BH3 only sensitizers. Bax translocates to the mitochondria where it becomes activated by BH3 only activators and forms a Bax pore which leads to MOMP and Cytochrome C release. Some elements of the figure were produced using Servier Medical art image bank (www.servier.com).

Bcl-xL retrotranslocation was later on analyzed by FLIP, using HCT116 cells (knocked out for *bax* and *bak* genes) expressing GFP-Bcl-xL. Apparently, in the absence of Bax, Bcl-xL localized predominantly to the mitochondria, slowly translocating back to the cytosol. However, the overexpression of Bax accelerated Bcl-xL translocation suggesting that both proteins interact at the level of the mitochondria and retro-translocate to the cytosol as a heterocomplex, which eventually dissociates (Figure 2). Of note, the overexpression of Bcl-2 and Mcl-1 accelerated Bax retrotranslocation in a similar manner to that of Bcl-xL. Thus, the retrotranslocation of Bax into the cytosol caused by apoptosis inhibitors may account for the observed cytosolic distribution of Bax in healthy cells (Edlich et al., 2011). Shortly after, Todt and colleagues showed that Bcl-xL C-terminal residues are important for Bax retrotranslocation, which is itself preceded by a conformational change unmasking Bax BH3 domain (Todt et al., 2013). Indeed, Bax retrotranslocation appears to depend on two types of interactions: (1) recognition of the Bax BH3 domain by the hydrophobic groove of Bcl-xL and (2) binding of Bcl-xL membrane anchor to Bax. The proposed model suggests that “Bax is the freight and Bcl-xL is the carrier for shuttling from the mitochondria into the cytoplasm.”

In 2015, the same team undertook a detailed comparison of Bak and Bax translocation mechanisms (Todt et al., 2015). They found that Bak is actually present in the cytosol and, using FLIP measurements, that Bcl-xL is responsible for Bak retrotranslocation from mitochondria to the cytosol. Overexpression of Mcl-1, but not Bcl-2, was found to accelerate this shuttling, as well as Bax. Thus, the same retrotranslocation process appears to take place for both Bax and Bak, Bax/Bak BH3 domain interaction with the hydrophobic groove of Bcl-xL being critical. Furthermore, the differential localization of Bak in the tissues might partly result from different Bak shuttling rates.

In fact, shuttling rates seem to depend more on the hydrophobicity rather than on the sequence of the TM domain. Using Bax and Bak proteins with swapped TM domains, Todt and colleagues demonstrated that increased Bax retrotranslocation to the cytosol protects the cells from Bax activation. Actually, not only Bax and Bak, but also MOM-integrated Bcl-2 proteins such as Bcl-xL, seem to shuttle similarly.

Although being predominantly mitochondrial, Bak leads to apoptosis only in the presence of apoptotic stimuli (Todt et al., 2015). On the other hand, when activated, Bax cannot be translocated anymore from the MOM to the cytosol (Edlich et al., 2011). Therefore, under non-apoptotic circumstances, Bax-dependent MOMP is blocked due to increased Bax retro-translocation to the cytosol, further reducing the time spent at the level of the MOM in order to prevent Bax activation (Edlich et al., 2011; Todt et al., 2015).

## Bax and bak trafficking through peroxisome/lysosome pathways

Although Bax and Bak shuttle between the cytosol and the mitochondria, it remains unclear if this process could occur between the mitochondria and the ER. Indeed both proteins were shown to be ER-resident where they control ER-mediated Ca^2+^ release and apoptosis induction (Scorrano et al., 2003; Zong et al., 2003). As a matter of fact, mitochondria and ER are continually moving along the cytoskeleton and undergo morphological changes *via* processes like membrane fission, fusion, degradation and renewal. In this regard, peroxisomes play an important role in ER-mitochondria protein exchanges. Indeed mitochondria-derived vesicles (MDVs) were shown to transport phospholipids and proteins from the mitochondria to the peroxisomes (Braschi et al., 2010) whereas peroxisomal proteins may be produced on membrane-bound polysomes of the ER and then transported to the peroxisomes (Dimitrov et al., 2013). Furthermore, it was recently shown that peroxisomes could also arise “*de novo”* from the fusion of organelle-derived membrane vesicles from the ER and mitochondria, highlighting the dynamic interplay between these organelles (Sugiura et al., 2017). Interestingly, some pro-apoptotic proteins, including Bak, were shown to localize to peroxisomes. This was found to occur when Bak could no longer interact with VDAC2 at the MOM (Cheng et al., 2003; Roy et al., 2009; Hosoi et al., 2017). Moreover, loss of VDAC2 diverts Bak into the peroxisomes, revealing a new activity for Bak in the control of peroxisome membrane integrity and the release of soluble peroxisomal matrix proteins (Hosoi et al., 2017).

**Figure 3:**
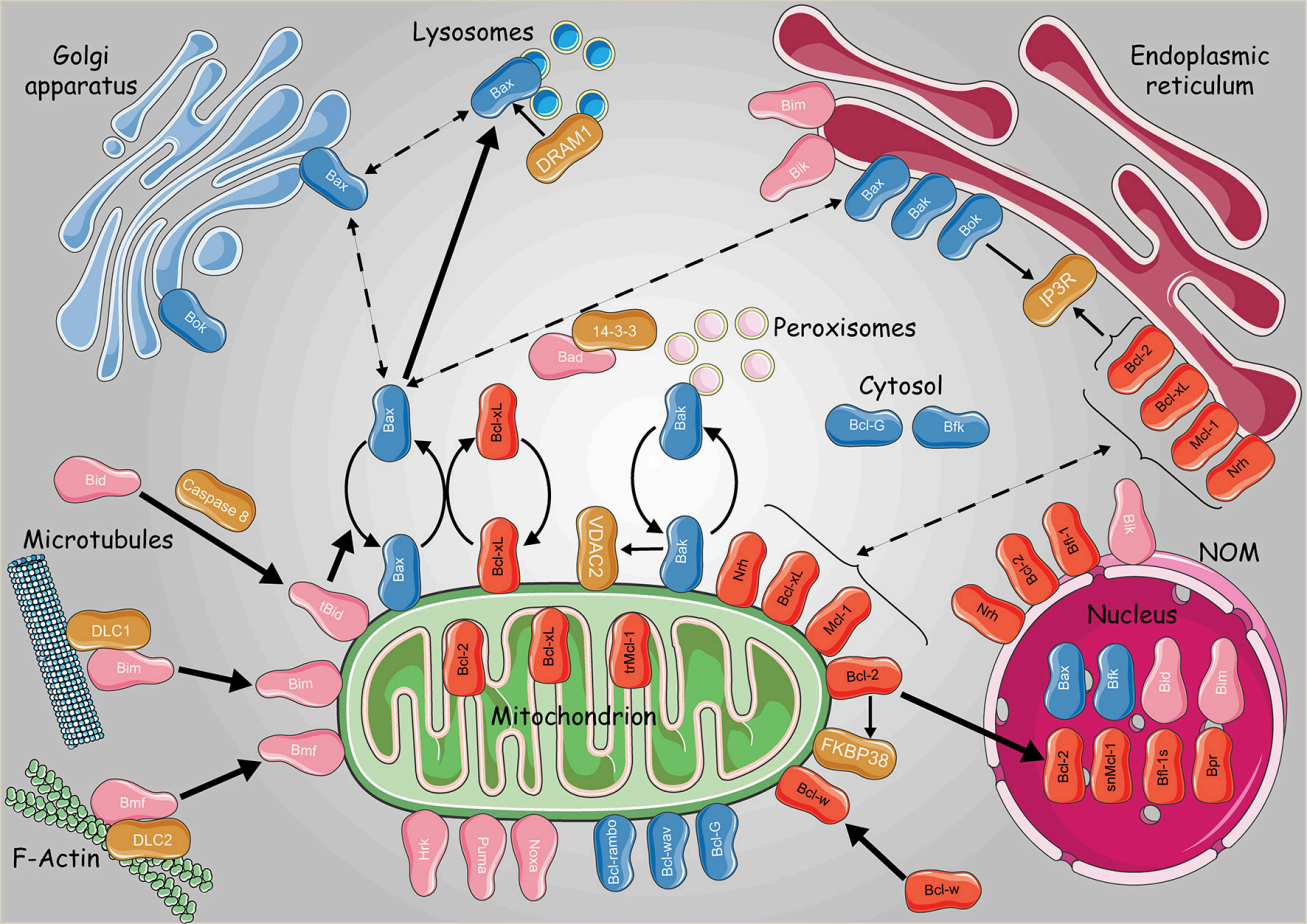
Subcellular dynamics of the Bcl-2 family of proteins. Schematic representation of the intracellular localizations and dynamics of anti-apoptotic (in red) pro-apoptotic multidomain (in blue) and BH3 only proteins (pink). Non Bcl-2 homologs were presented with dark orange boxes. Continuous and bold lines represent protein translocation in physiological and stress conditions, respectively. Dashed lines represent hypothetical subcellular translocation. trMcl-1, truncated Mcl-1; snMcl-1, shortened nuclear Mcl-1; Bfl-1s, Bfl-1 short isoform. Some elements of the figure were produced using Servier Medical art image bank (www.servier.com).

Bcl-2 proteins may also navigate between the mitochondria and the Golgi apparatus. Indeed pro-apoptotic Bax and Bok were found to be addressed to this later compartment (Figure 3). Bok was suggested to act upstream of Bax and Bak, controlling the communication between ER-Golgi compartments and the mitochondria through apoptotic signals. In fact, Echeverry and colleagues observed that Bok, when overexpressed, contributed to the fragmentation of the Golgi and the ER before the activation of caspases. On the other hand, Bok silencing resulted in Golgi disassembly under stressful conditions, thus boosting ER stress and subsequently activating BH3-only proteins and apoptosis (Echeverry et al., 2013).

Bax localization to the Golgi was observed in human embryonic stem cells (Dumitru et al., 2012). In fact, using the 6A7 antiBax monoclonal and the TGN46 trans-Golgi network protein, Dumitru and colleagues demonstrated that Bax locates in the *trans* Golgi compartment in a specific manner, being totally absent from the *cis* compartment. In addition, after DNA damage, Bax seemed to translocate from the Golgi to the mitochondria in a p53-dependent manner (Dumitru et al., 2012). Another study performed by Guan and colleagues showed that the lysosomes are also involved in Bax-mediated apoptosis, revealing a novel crosstalk between autophagy and apoptosis through DRAM1 (DNA damage-regulated autophagy modulator 1) (Guan et al., 2015). Actually, Bax affects the permeability of the lysosomes and the rate of lysosomal cathepsins release (Feldstein et al., 2006; Oberle et al., 2010). Furthermore, upon treatment with the mitochondrial complex II inhibitor 3-nitropropionic acid (3NP), DRAM1 and Bax expression levels were increased, contributing to lysosomal Bax relocalization in a DRAM1-dependent manner, causing the release of lysosomal cathepsin B as well as Bid cleavage (Guan et al., 2015). Accordingly, the next questions were: “How does Bax migrate in all these cellular compartments? Is there a sort of retrotranslocation similar to that of cytosol-mitochondria?”

The lysosomes are formed by the fusion of transport vesicles budded from the *trans* Golgi network with endosomes, which contain molecules taken up by endocytosis at the level of the plasma membrane. On this basis, the presence of Bax in the *trans* Golgi compartments can be clarified by suggesting that lysosomes could act as transport vesicles of Bax. However, this latter issue needs further investigations.

Thus, two hypotheses are open regarding the intracellular dynamics of Bcl-2 proteins: either (1) each protein has a specific localization signal that targets to a specific compartment, or (2) the same protein can *a priori* locate in different compartments, inter-organelle translocation being ensured by dedicated carriers.

Actually, both hypotheses are non-mutually exclusive. Intracellular vesicles—along with protein-protein interactions—could be responsible for the transit of Bcl-2 proteins inside the cell as suggested in the case of peroxisomal Bak. On the other hand, protein-protein interactions and stress signals can also trigger the unmasking of specific sequences addressing the protein to a specific compartment as in the case of Bcl-xL and Bax.

## Moonlighting functions of the Bcl-2 proteins in respect to their subcellular localizations

### Interactions with ER Ca^2+^ channels

Bcl-2 proteins are obviously able to shuttle between different cellular compartments. However, until now, it has been generally admitted that their primary role is to control apoptosis at the level of the mitochondria. After more than 30 years of research, it is now acknowledged that Bcl-2 proteins play multiple non-canonical roles beyond apoptosis (Bonneau et al., 2013). They appear to be able do so by means of their various subcellular localizations.

Besides their mitochondrial localization, Bcl-2 proteins can also localize to the ER. At the level of this later compartment, Bcl-2 family members regulate apoptosis through the control of Ca^2+^ fluxes through the ER-mitochondria contact sites. Indeed, mitochondria constantly take up Ca^2+^ ions, stimulating biogenesis and ATP production. They also act as an intracellular Ca^2+^ buffer due to their rapid uptake of Ca^2+^ ions, when massively released from the ER. Excessive accumulation of mitochondrial Ca^2+^ may lead to mitochondria swelling and cell death. Bcl-2 proteins control Ca^2+^ trafficking through direct interactions with ER Ca^2+^ transporters. For example, Bcl-2 has long been studied for its interaction with IP3R. This interaction, either with the BH4 domain of Bcl-2 (Rong et al., 2008, 2009) or with its trans-membrane domain (Ivanova et al., 2016), inhibits ER Ca^2+^ release. On the other hand, the hydrophobic pocket of Bcl-xL (Yang et al., 2016) sensitizes IP3R to low levels of IP3, leading to the opening of the channel (White et al., 2005).

Using the zebrafish model, we demonstrated that a Bcl-2 homolog, namely Nrz (Nr-13 ortholog in zebrafish), is critical during the early stages of zebrafish development (Arnaud et al., 2006). In zebrafish Nrz protein possesses a dual subcellular localization both at the ER and the mitochondria. Nrz silencing causes premature gastrulation arrest followed by detachment of the entire blastomeres from the yolk sac (Popgeorgiev et al., 2011). By performing a series of time lapse and confocal microscopy experiments, we demonstrated that this phenotype is due to the premature formation of the actin-myosin contractile ring, a supramolecular structure, which squeezes the yolk cell at the level of the margin and cuts the embryo in half. Furthermore, we showed that the ER-resident Nrz, but not mitochondrial-resident Nrz, was critical for gastrulation progression. We showed that, at the level of the ER, Nrz interacts with IP3R1 Ca^2+^ channels (Popgeorgiev et al., 2011) and regulates the time course of the release of Ca^2+^ into the yolk sac by competing with IP3 (Bonneau et al., 2014). In addition, we established that, by doing so, Nrz controls the formation of the contractile actin-myosin ring *via* a Calmodulin-myosin light chain kinase (MLCK)-dependent pathway. This process seems evolutionary conserved since Nrh, the human Nrz ortholog, also prevents IP3R opening. Interestingly, Nrh anti-apoptotic activity was reported to be under the control of inositol 1,4,5-trisphosphate (IP3) receptor-binding protein (IRBIT), another partner of IP3R (Bonneau et al., 2016). In fact, Nrh may indirectly prevent apoptosis by controlling ER-stress. Indeed, there is evidence that Nrh acts upstream of the Unfolded Protein Response (UPR) by inhibiting ERCa^2+^ release through IP3R closure (Nougarede et al., 2018).

On the other hand, regarding cell death accelerators, the only pro-apoptotic protein reported to directly interact with IP3R is Bok. This occurs through the BH4 domain of Bok which binds the coupling domain of IP3R1 and IP3R2. However, this interaction does not affect Ca^2+^release, although it protects the channel from proteolytic cleavage (Schulman et al., 2013).

### Bcl-2 proteins and the nucleus

Numerous Bcl-2-related proteins were found to localize to the nucleus, contributing to vital processes. For example, Gross and colleagues reported that a fraction of the BH3-only protein Bid located to the nucleus, plays a role in the DNA damage response (Kamer et al., 2005; Zinkel et al., 2005). In fact, the ATM and ATR kinases are activated following DNA damage, causing cell cycle arrest and subsequent DNA repair or apoptosis. Interestingly, one of the targets of ATM is nuclear Bid, which is critical for cell cycle arrest at the S phase and apoptosis induction (Zinkel et al., 2005). It was proposed that, following DNA damage, Bid could be transported to the nucleus as part of a protein complex since it lacks an obvious nuclear localization signal. Accordingly, Bid might either help stabilize the complex or facilitate subsequent enzymatic steps. In this respect, Bid phosphorylation is critical for maintaining genomic stability at S phase check points. Overall, these data suggest that Bid acts as a mediator between apoptosis and cell cycle regulation during S phase.

Regarding Bcl-2, much evidence support existing interactions—either direct or indirect—with transcription factors. Indeed, de Moissac and colleagues reported an interaction between Bcl-2 and IκBα, the cytoplasmic inhibitor of the ubiquitously expressed transcription factor Nuclear Factor κB (NFκB). They could show that Bcl-2 facilitates IκBα degradation, activating in turn NFκB (de Moissac et al., 1998). Another study provided direct evidence that NFκB transcriptionally regulates *bcl2* gene expression, directly linking the TNF-α/NFκB signaling pathway to *bcl2* expression in human prostate carcinoma cells (Catz and Johnson, 2001). This regulation was later shown to control cell invasiveness in estrogen receptor alpha (ERα)-negative breast cancer cells (Wang et al., 2007). A recent study delineated the regulation of gene transcription by extra-mitochondrial Bcl-2 proteins, notably Bcl-2, Bcl-xL, and Mcl-1. In fact, these proteins engage a BH domain found in SUFU, the tumor suppressor and antagonist of GLI DNA-binding proteins. By doing so, they promote SUFU turnover, impede its interaction with GLI and allow the expression of GLI target genes (including Bcl-2, Bcl-xL and Mcl-1) thus prompting the survival and growth of cancer cells (Wu et al., 2017).

## Concluding remarks

Bcl-2 family proteins are arguably one of the main regulators of the mitochondrial outer membrane permeability. Since their discovery it became abundantly clear that the vast majority of these proteins can localize in multiple subcellular compartments where they not only remotely control MOMP but also participate in additional non apoptotic functions (Bonneau et al., 2013). Although structurally related, Bcl-2 family proteins present important differences in their subcellular localizations, which were attributed to subtle intramolecular variations of their TM amino acid composition. These observations led to a rather predeterministic view of Bcl-2 family intracellular distribution in which once synthetized, these proteins were directly targeted to precise subcellular compartments (Kaufmann et al., 2003). However, more recent data proposed that at least part of the Bcl-2 family can translocate and change their subcellular localizations depending on the cellular status and apoptotic stimuli (Edlich et al., 2011; Todt et al., 2013, 2015). How exactly Bcl-2 proteins navigate through the intracellular membrane network remains currently unexplained. Two recent reports demonstrated that at least the pro-apoptotic Bax and Bak can localize to lysosomes and peroxisomes respectively (Oberle et al., 2010; Guan et al., 2015; Fujiki et al., 2017). Peroxisomes are critical in ER-mitochondria protein exchanges and lysosomes are derived from ER and Golgi membranes. Thus, it is tempting to speculate that Bcl-2 proteins trafficking may occur through vesicular transport inside the cell. However, further studies are expected to reveal the exact mechanisms of Bcl-2 family subcellular dynamics.

Is there a difference between subcellular distribution of Bcl-2 proteins between normal and cancer cells and if so, does this difference contribute to cancer progression or chemoresistance represents another open question. Early studies suggested that Bcl-2 is similarly distributed in normal peripheral blood lymphocytes and CLL cells (de Jong et al., 1994). However, more recently, differences in the intracellular distribution have been observed for individual Bcl-2 homologs. For example, nuclear localization of Bcl-2 and Bcl-xL was observed in various cancer cells including breast cancer, endometrial carcinoma, squamous cell carcinoma, and astrocytoma (Chan et al., 1995; Mosnier et al., 1996; Choi et al., 2016). Interestingly, nuclear Bcl-xL localization was found to correlate with epithelial–mesenchymal transition and increased metastasis formation. Thus, it would be of interest to further analyse the molecular mechanisms behind Bcl-2 intracellular distribution in tumor cells (Choi et al., 2016).

Globally, and to conclude, this review provides new insights into the multiple subcellular localizations of Bcl-2 proteins and the way-too-long masked importance of their extra-mitochondrial distribution.

## Author contributions

NP, LJ, and GG wrote the manuscript and approved it for publication.

### Conflict of interest statement

The authors declare that the research was conducted in the absence of any commercial or financial relationships that could be construed as a potential conflict of interest.

## References

[B1] Akao Y. Otsuki Y. Kataoka S. Ito Y. Tsujimoto Y. (1994). Multiple subcellular localization of bcl-2: detection in nuclear outer membrane, endoplasmic reticulum membrane, and mitochondrial membranes. Cancer Res. 54, 2468–2471. 8162596

[B2] Andreu-Fernández V. García-Murria M. J. Bañó-Polo M. Martin J. Monticelli L. Orzáez M.. (2016). The C-terminal domains of apoptotic BH3-only proteins mediate their insertion into distinct biological membranes. J. Biol. Chem. 291, 25207–25216. 10.1074/jbc.M116.733634 27758854 PMC5122786

[B3] Aouacheria A. Arnaud E. Venet S. Lalle P. Gouy M. Rigal D.. (2001). Nrh, a human homologue of Nr-13 associates with Bcl-Xs and is an inhibitor of apoptosis. Oncogene 20, 5846–5855. 10.1038/sj.onc.1204740 11593390

[B4] Arnaud E. Ferri K. F. Thibaut J. Haftek-Terreau Z. Aouacheria A. Le Guellec D.. (2006). The zebrafish bcl-2 homologue Nrz controls development during somitogenesis and gastrulation via apoptosis-dependent and -independent mechanisms. Cell Death Differ. 13, 1128–1137. 10.1038/sj.cdd.4401797 16282981

[B5] Barboule N. Demeter K. Benmeradi N. Larminat F. (2009). Bcl-2 is an integral component of mitotic chromosomes. Cell Biol. Int. 33, 572–577. 10.1016/j.cellbi.2009.02.012 19269343

[B6] Barboule N. Truchet I. Valette A. (2005). Localization of phosphorylated forms of Bcl-2 in mitosis: co-localization with Ki-67 and nucleolin in nuclear structures and on mitotic chromosomes. Cell Cycle 4, 590–596. 10.4161/cc.4.4.1587 15876860

[B7] Bonneau B. Ando H. Kawaai K. Hirose M. Takahashi-Iwanaga H. Mikoshiba K. (2016). IRBIT controls apoptosis by interacting with the Bcl-2 homolog, Bcl2l10, and by promoting ER-mitochondria contact. eLife 5:e19896. 10.7554/eLife.19896 27995898 PMC5173324

[B8] Bonneau B. Nougarède A. Prudent J. Popgeorgiev N. Peyriéras N. Rimokh R.. (2014). The Bcl-2 homolog Nrz inhibits binding of IP3 to its receptor to control calcium signaling during zebrafish epiboly. Sci. Signal. 7:ra14. 10.1126/scisignal.2004480 24518293

[B9] Bonneau B. Prudent J. Popgeorgiev N. Gillet G. (2013). Non-apoptotic roles of Bcl-2 family: the calcium connection. Biochim. Biophys. Acta 1833, 1755–1765. 10.1016/j.bbamcr.2013.01.021 23360981

[B10] Braschi E. Goyon V. Zunino R. Mohanty A. Xu L. McBride H. M. (2010). Vps35 mediates vesicle transport between the mitochondria and peroxisomes. Curr. Biol. 20, 1310–1315. 10.1016/j.cub.2010.05.066 20619655

[B11] Cartron P. F. Priault M. Oliver L. Meflah K. Manon S. Vallette F. M. (2003). The N-terminal end of Bax contains a mitochondrial-targeting signal. J. Biol. Chem. 278, 11633–11641. 10.1074/jbc.M208955200 12529375

[B12] Catz S. D. Johnson J. L. (2001). Transcriptional regulation of bcl-2 by nuclear factor kappa B and its significance in prostate cancer. Oncogene 20, 7342–7351. 10.1038/sj.onc.1204926 11704864

[B13] Chan W. K. Mole M. M. Levison D. A. Ball R. Y. Lu Q. L. Patel K.. (1995). Nuclear and cytoplasmic bcl-2 expression in endometrial hyperplasia and adenocarcinoma. J. Pathol. 177, 241–246. 10.1002/path.1711770305 8551385

[B14] Cheng E. H. Sheiko T. V. Fisher J. K. Craigen W. J. Korsmeyer S. J. (2003). VDAC2 inhibits BAK activation and mitochondrial apoptosis. Science 301, 513–517. 10.1126/science.1083995 12881569

[B15] Chen-Levy Z. Nourse J. Cleary M. L. (1989). The bcl-2 candidate proto-oncogene product is a 24-kilodalton integral-membrane protein highly expressed in lymphoid cell lines and lymphomas carrying the t(14;18) translocation. Mol. Cell. Biol. 9, 701–710. 10.1128/MCB.9.2.701 2651903 PMC362647

[B16] Choi S. Chen Z. Tang L. H. Fang Y. Shin S. J. Panarelli N. C.. (2016). Bcl-xL promotes metastasis independent of its anti-apoptotic activity. Nat. Commun. 7:10384. 10.1038/ncomms10384 26785948 PMC4735924

[B17] Cleary M. L. Smith S. D. Sklar J. (1986). Cloning and structural analysis of cDNAs for bcl-2 and a hybrid bcl-2/immunoglobulin transcript resulting from the t(14;18) translocation. Cell 47, 19–28. 10.1016/0092-8674(86)90362-4 2875799

[B18] de Jong D. Prins F. A. Mason D. Y. Reed J. C. van Ommen G. B. Kluin P. M. (1994). Subcellular localization of the bcl-2 protein in malignant and normal lymphoid cells. Cancer Res. 54, 256–260. 8261449

[B19] Delbridge A. R. Grabow S. Strasser A. Vaux D. L. (2016). Thirty years of BCL-2: translating cell death discoveries into novel cancer therapies. Nat. Rev. Cancer 16, 99–109. 10.1038/nrc.2015.17 26822577

[B20] de Moissac D. Mustapha S. Greenberg A. H. Kirshenbaum L. A. (1998). Bcl-2 activates the transcription factor NFκB through the degradation of the cytoplasmic inhibitor IκBα. J. Biol. Chem. 273, 23946–23951. 10.1074/jbc.273.37.23946 9727009

[B21] Desagher S. Osen-Sand A. Nichols A. Eskes R. Montessuit S. Lauper S.. (1999). Bid-induced conformational change of Bax is responsible for mitochondrial cytochrome c release during apoptosis. J. Cell Biol. 144, 891–901. 10.1083/jcb.144.5.891 10085289 PMC2148190

[B22] Dimitrov L. Lam S. K. Schekman R. (2013). The role of the endoplasmic reticulum in peroxisome biogenesis. Cold Spring Harb. Perspect. Biol. 5:a013243. 10.1101/cshperspect.a013243 23637287 PMC3632059

[B23] Dumitru R. Gama V. Fagan B. M. Bower J. J. Swahari V. Pevny L. H.. (2012). Human embryonic stem cells have constitutively active Bax at the Golgi and are primed to undergo rapid apoptosis. Mol. Cell 46, 573–583. 10.1016/j.molcel.2012.04.002 22560721 PMC3372694

[B24] Echeverry N. Bachmann D. Ke F. Strasser A. Simon H. U. Kaufmann T. (2013). Intracellular localization of the BCL-2 family member BOK and functional implications. Cell Death Differ. 20, 785–799. 10.1038/cdd.2013.10 23429263 PMC3647236

[B25] Edlich F. Banerjee S. Suzuki M. Cleland M. M. Arnoult D. Wang C.. (2011). Bcl-x(L) retrotranslocates Bax from the mitochondria into the cytosol. Cell 145, 104–116. 10.1016/j.cell.2011.02.034 21458670 PMC3070914

[B26] Er E. Lalier L. Cartron P. F. Oliver L. Vallette F. M. (2007). Control of Bax homodimerization by its carboxyl terminus. J. Biol. Chem. 282, 24938–24947. 10.1074/jbc.M703823200 17556360

[B27] Feldstein A. E. Werneburg N. W. Li Z. Bronk S. F. Gores G. J. (2006). Bax inhibition protects against free fatty acid-induced lysosomal permeabilization. Am. J. Physiol. Gastrointest. Liver Physiol. 290, G1339–G1346. 10.1152/ajpgi.00509.2005 16484678 PMC3056273

[B28] Fujiki Y. Miyata N. Mukai S. Okumoto K. Cheng E. H. (2017). BAK regulates catalase release from peroxisomes. Mol. Cell. Oncol. 4:e1306610. 10.1080/23723556.2017.1306610 28616584 PMC5462519

[B29] George N. M. Evans J. J. Luo X. (2007). A three-helix homo-oligomerization domain containing BH3 and BH1 is responsible for the apoptotic activity of Bax. Genes Dev. 21, 1937–1948. 10.1101/gad.1553607 17671092 PMC1935031

[B30] Germain M. Mathai J. P. Shore G. C. (2002). BH-3-only BIK functions at the endoplasmic reticulum to stimulate cytochrome c release from mitochondria. J. Biol. Chem. 277, 18053–18060. 10.1074/jbc.M201235200 11884414

[B31] Guan J. J. Zhang X. D. Sun W. Qi L. Wu J. C. Qin Z. H. (2015). DRAM1 regulates apoptosis through increasing protein levels and lysosomal localization of BAX. Cell Death Dis. 6:e1624. 10.1038/cddis.2014.546 25633293 PMC4669745

[B32] Hockenbery D. Nuñez G. Milliman C. Schreiber R. D. Korsmeyer S. J. (1990). Bcl-2 is an inner mitochondrial membrane protein that blocks programmed cell death. Nature 348, 334–336. 10.1038/348334a0 2250705

[B33] Hosoi K. I. Miyata N. Mukai S. Furuki S. Okumoto K. Cheng E. H.. (2017). The VDAC2-BAK axis regulates peroxisomal membrane permeability. J. Cell Biol. 216, 709–722. 10.1083/jcb.201605002 28174205 PMC5350511

[B34] Hsu Y. T. Wolter K. G. Youle R. J. (1997). Cytosol-to-membrane redistribution of Bax and Bcl-X(L) during apoptosis. Proc. Natl. Acad. Sci. U.S.A. 94, 3668–3672. 10.1073/pnas.94.8.3668 9108035 PMC20498

[B35] Ivanova H. Ritaine A. Wagner L. Luyten T. Shapovalov G. Welkenhuyzen K. (2016). The trans-membrane domain of Bcl-2α, but not its hydrophobic cleft, is a critical determinant for efficient IP3 receptor inhibition. Oncotarget 7, 55704–55720. 10.18632/oncotarget.11005 27494888 PMC5342447

[B36] Jeong S. Y. Gaume B. Lee Y. J. Hsu Y. T. Ryu S. W. Yoon S. H.. (2004). Bcl-x(L) sequesters its C-terminal membrane anchor in soluble, cytosolic homodimers. EMBO J. 23, 2146–2155. 10.1038/sj.emboj.7600225 15131699 PMC424420

[B37] Kamer I. Sarig R. Zaltsman Y. Niv H. Oberkovitz G. Regev L.. (2005). Proapoptotic BID is an ATM effector in the DNA-damage response. Cell 122, 593–603. 10.1016/j.cell.2005.06.014 16122426

[B38] Kaufmann T. Schlipf S. Sanz J. Neubert K. Stein R. Borner C. (2003). Characterization of the signal that directs Bcl-x(L), but not Bcl-2, to the mitochondrial outer membrane. J. Cell Biol. 160, 53–64. 10.1083/jcb.200210084 12515824 PMC2172731

[B39] Kim H. Tu H. C. Ren D. Takeuchi O. Jeffers J. R. Zambetti G. P.. (2009). Stepwise activation of BAX and BAK by tBID, BIM, and PUMA initiates mitochondrial apoptosis. Mol. Cell 36, 487–499. 10.1016/j.molcel.2009.09.030 19917256 PMC3163439

[B40] Krajewski S. Tanaka S. Takayama S. Schibler M. J. Fenton W. Reed J. C. (1993). Investigation of the subcellular distribution of the bcl-2 oncoprotein: residence in the nuclear envelope, endoplasmic reticulum, and outer mitochondrial membranes. Cancer Res. 53, 4701–4714. 8402648

[B41] Kuwana T. Bouchier-Hayes L. Chipuk J. E. Bonzon C. Sullivan B. A. Green D. R.. (2005). BH3 domains of BH3-only proteins differentially regulate Bax-mediated mitochondrial membrane permeabilization both directly and indirectly. Mol. Cell 17, 525–535. 10.1016/j.molcel.2005.02.003 15721256

[B42] Letai A. Bassik M. C. Walensky L. D. Sorcinelli M. D. Weiler S. Korsmeyer S. J. (2002). Distinct BH3 domains either sensitize or activate mitochondrial apoptosis, serving as prototype cancer therapeutics. Cancer Cell 2, 183–192. 10.1016/S1535-6108(02)00127-7 12242151

[B43] McNally M. A. Soane L. Roelofs B. A. Hartman A. L. Hardwick J. M. (2013). The N-terminal helix of Bcl-xL targets mitochondria. Mitochondrion 13, 119–124. 10.1016/j.mito.2013.01.004 23333404 PMC3650083

[B44] Monaco G. Beckers M. Ivanova H. Missiaen L. Parys J. B. De Smedt H.. (2012a). Profiling of the Bcl-2/Bcl-X(L)-binding sites on type 1 IP(3) receptor. Biochem. Biophys. Res. Commun. 428, 31–35. 10.1016/j.bbrc.2012.10.002 23058917

[B45] Monaco G. Decrock E. Akl H. Ponsaerts R. Vervliet T. Luyten T.. (2012b). Selective regulation of IP_3_-receptor-mediated Ca^2+^ signaling and apoptosis by the BH4 domain of Bcl-2 versus Bcl-Xl. Cell Death Differ. 19, 295–309. 10.1038/cdd.2011.97 21818117 PMC3263504

[B46] Morishima N. Nakanishi K. Tsuchiya K. Shibata T. Seiwa E. (2004). Translocation of Bim to the endoplasmic reticulum (ER) mediates ER stress signaling for activation of caspase-12 during ER stress-induced apoptosis. J. Biol. Chem. 279, 50375–50381. 10.1074/jbc.M408493200 15452118

[B47] Mosnier J. F. Perret A. G. Brunon J. Boucheron S. (1996). Expression of the bcl-2 oncoprotein in meningiomas. Am. J. Clin. Pathol. 106, 652–659. 10.1093/ajcp/106.5.652 8929477

[B48] Nakai M. Takeda A. Cleary M. L. Endo T. (1993). The bcl-2 protein is inserted into the outer membrane but not into the inner membrane of rat liver mitochondria *in vitro*. Biochem. Biophys. Res. Commun. 196, 233–239. 10.1006/bbrc.1993.2239 8216296

[B49] Nguyen M. Millar D. G. Yong V. W. Korsmeyer S. J. Shore G. C. (1993). Targeting of Bcl-2 to the mitochondrial outer membrane by a COOH-terminal signal anchor sequence. J. Biol. Chem. 268, 25265–25268. 8244956

[B50] Nougarede A. Popgeorgiev N. Kassem L. Omarjee S. Borel S. Mikaelian I.. (2018). Breast cancer targeting through inhibition of the endoplasmic reticulum-based apoptosis regulator Nrh/BCL2L10. Cancer Res. [Epub ahead of print]. 10.1158/0008-5472.CAN-17-0846 29330143

[B51] Oberle C. Huai J. Reinheckel T. Tacke M. Rassner M. Ekert P. G.. (2010). Lysosomal membrane permeabilization and cathepsin release is a Bax/Bak-dependent, amplifying event of apoptosis in fibroblasts and monocytes. Cell Death Differ. 17, 1167–1178. 10.1038/cdd.2009.214 20094062

[B52] Perciavalle R. M. Stewart D. P. Koss B. Lynch J. Milasta S. Bathina M.. (2012). Anti-apoptotic MCL-1 localizes to the mitochondrial matrix and couples mitochondrial fusion to respiration. Nat. Cell Biol. 14, 575–583. 10.1038/ncb2488 22544066 PMC3401947

[B53] Popgeorgiev N. Bonneau B. Ferri K. F. Prudent J. Thibaut J. Gillet G. (2011). The apoptotic regulator Nrz controls cytoskeletal dynamics via the regulation of Ca2+ trafficking in the zebrafish blastula. Dev. Cell 20, 663–676. 10.1016/j.devcel.2011.03.016 21571223

[B54] Portier B. P. Taglialatela G. (2006). Bcl-2 localized at the nuclear compartment induces apoptosis after transient overexpression. J. Biol. Chem. 281, 40493–40502. 10.1074/jbc.M606181200 17090549

[B55] Quinn L. Coombe M. Mills K. Daish T. Colussi P. Kumar S.. (2003). Buffy, a *Drosophila* Bcl-2 protein, has anti-apoptotic and cell cycle inhibitory functions. EMBO J. 22, 3568–3579. 10.1093/emboj/cdg355 12853472 PMC165625

[B56] Rong Y. P. Aromolaran A. S. Bultynck G. Zhong F. Li X. McColl K.. (2008). Targeting Bcl-2-IP3 receptor interaction to reverse Bcl-2's inhibition of apoptotic calcium signals. Mol. Cell 31, 255–265. 10.1016/j.molcel.2008.06.014 18657507 PMC3660092

[B57] Rong Y. P. Barr P. Yee V. C. Distelhorst C. W. (2009). Targeting Bcl-2 based on the interaction of its BH4 domain with the inositol 1,4,5-trisphosphate receptor. Biochim. Biophys. Acta 1793, 971–978. 10.1016/j.bbamcr.2008.10.015 19056433 PMC3674874

[B58] Roy S. S. Ehrlich A. M. Craigen W. J. Hajnóczky G. (2009). VDAC2 is required for truncated BID-induced mitochondrial apoptosis by recruiting BAK to the mitochondria. EMBO Rep. 10, 1341–1347. 10.1038/embor.2009.219 19820692 PMC2799216

[B59] Schulman J. J. Wright F. A. Kaufmann T. Wojcikiewicz R. J. (2013). The Bcl-2 protein family member Bok binds to the coupling domain of inositol 1,4,5-trisphosphate receptors and protects them from proteolytic cleavage. J. Biol. Chem. 288, 25340–25349. 10.1074/jbc.M113.496570 23884412 PMC3757198

[B60] Scorrano L. Oakes S. A. Opferman J. T. Cheng E. H. Sorcinelli M. D. Pozzan T.. (2003). BAX and BAK regulation of endoplasmic reticulum Ca2+: a control point for apoptosis. Science 300, 135–139. 10.1126/science.1081208 12624178

[B61] Shirane M. Nakayama K. I. (2003). Inherent calcineurin inhibitor FKBP38 targets Bcl-2 to mitochondria and inhibits apoptosis. Nat. Cell Biol. 5, 28–37. 10.1038/ncb894 12510191

[B62] Sugiura A. Mattie S. Prudent J. McBride H. M. (2017). Newly born peroxisomes are a hybrid of mitochondrial and ER-derived pre-peroxisomes. Nature 542, 251–254. 10.1038/nature21375 28146471

[B63] Tan F. J. Fire A. Z. Hill R. B. (2007). Regulation of apoptosis by *C. elegans* CED-9 in the absence of the C-terminal transmembrane domain. Cell Death Differ. 14, 1925–1935. 10.1038/sj.cdd.4402215 17703231 PMC3047747

[B64] Todt F. Cakir Z. Reichenbach F. Emschermann F. Lauterwasser J. Kaiser A.. (2015). Differential retrotranslocation of mitochondrial Bax and Bak. EMBO J. 34, 67–80. 10.15252/embj.201488806 25378477 PMC4291481

[B65] Todt F. Cakir Z. Reichenbach F. Youle R. J. Edlich F. (2013). The C-terminal helix of Bcl-x(L) mediates Bax retrotranslocation from the mitochondria. Cell Death Differ. 20, 333–342. 10.1038/cdd.2012.131 23079612 PMC3554327

[B66] Tsujimoto Y. Finger L. R. Yunis J. Nowell P. C. Croce C. M. (1984). Cloning of the chromosome breakpoint of neoplastic B cells with the t(14;18) chromosome translocation. Science 226, 1097–1099. 10.1126/science.6093263 6093263

[B67] Tsujimoto Y. Jaffe E. Cossman J. Gorham J. Nowell P. C. Croce C. M. (1985). Clustering of breakpoints on chromosome 11 in human B-cell neoplasms with the t(11;14) chromosome translocation. Nature 315, 340–343. 10.1038/315340a0 3923362

[B68] Valero J. G. Sancey L. Kucharczak J. Guillemin Y. Gimenez D. Prudent J.. (2011). Bax-derived membrane-active peptides act as potent and direct inducers of apoptosis in cancer cells. J. Cell Sci. 124, 556–564. 10.1242/jcs.076745 21245196 PMC3428271

[B69] Vaux D. L. Cory S. Adams J. M. (1988). Bcl-2 gene promotes haemopoietic cell survival and cooperates with c-myc to immortalize pre-B cells. Nature 335, 440–442. 10.1038/335440a0 3262202

[B70] Wang X. Belguise K. Kersual N. Kirsch K. H. Mineva N. D. Galtier F.. (2007). Oestrogen signalling inhibits invasive phenotype by repressing RelB and its target BCL2. Nat. Cell Biol. 9, 470–478. 10.1038/ncb1559 17369819 PMC2394707

[B71] White C. Li C. Yang J. Petrenko N. B. Madesh M. Thompson C. B.. (2005). The endoplasmic reticulum gateway to apoptosis by Bcl-X(L) modulation of the InsP3R. Nat. Cell Biol. 7, 1021–1028. 10.1038/ncb1302 16179951 PMC2893337

[B72] Wilfling F. Weber A. Potthoff S. Vögtle F. N. Meisinger C. Paschen S. A.. (2012). BH3-only proteins are tail-anchored in the outer mitochondrial membrane and can initiate the activation of Bax. Cell Death Differ. 19, 1328–1336. 10.1038/cdd.2012.9 22343714 PMC3392640

[B73] Wolter K. G. Hsu Y. T. Smith C. L. Nechushtan A. Xi X. G. Youle R. J. (1997). Movement of Bax from the cytosol to mitochondria during apoptosis. J. Cell Biol. 139, 1281–1292. 10.1083/jcb.139.5.1281 9382873 PMC2140220

[B74] Wu X. Zhang L. S. Toombs J. Kuo Y. C. Piazza J. T. Tuladhar R.. (2017). Extra-mitochondrial prosurvival BCL-2 proteins regulate gene transcription by inhibiting the SUFU tumour suppressor. Nat. Cell Biol. 19, 1226–1236. 10.1038/ncb3616 28945232 PMC5657599

[B75] Yang J. Vais H. Gu W. Foskett J. K. (2016). Biphasic regulation of InsP3 receptor gating by dual Ca^2+^ release channel BH3-like domains mediates Bcl-xL control of cell viability. Proc. Nat. Acad. Sci. U.S.A. 113, E1953–E1962. 10.1073/pnas.1517935113 26976600 PMC4822637

[B76] Yang T. Kozopas K. M. Craig R. W. (1995). The intracellular distribution and pattern of expression of Mcl-1 overlap with, but are not identical to, those of Bcl-2. J. Cell Biol. 128, 1173–1184. 10.1083/jcb.128.6.1173 7896880 PMC2120408

[B77] Youle R. J. Strasser A. (2008). The BCL-2 protein family: opposing activities that mediate cell death. Nat. Rev. Mol. Cell Biol. 9, 47–59. 10.1038/nrm2308 18097445

[B78] Zinkel S. S. Hurov K. E. Ong C. Abtahi F. M. Gross A. Korsmeyer S. J. (2005). A role for proapoptotic BID in the DNA-damage response. Cell 122, 579–591. 10.1016/j.cell.2005.06.022 16122425

[B79] Zong W. X. Li C. Hatzivassiliou G. Lindsten T. Yu Q. C. Yuan J.. (2003). Bax and Bak can localize to the endoplasmic reticulum to initiate apoptosis. J. Cell Biol. 162, 59–69. 10.1083/jcb.200302084 12847083 PMC2172724

